# LFSC: A linear fast semi-supervised clustering algorithm that integrates reference-bulk and single-cell transcriptomes

**DOI:** 10.3389/fgene.2022.1068075

**Published:** 2022-12-01

**Authors:** Qiaoming Liu, Yingjian Liang, Dong Wang, Jie Li

**Affiliations:** ^1^ School of Computer Science and Technology, Harbin Institute of Technology, Harbin, China; ^2^ Department of General Surgery, The First Affiliated Hospital of Harbin Medical University, Harbin, China; ^3^ Key Laboratory of Hepatosplenic Surgery, Ministry of Education, The First Affiliated Hospital of Harbin Medical University, Harbin, China

**Keywords:** single-cell RNA-seq, bulk RNA-seq, anchor graph, data integration, clustering

## Abstract

The identification of cell types in complex tissues is an important step in research into cellular heterogeneity in disease. We present a linear fast semi-supervised clustering (LFSC) algorithm that utilizes reference samples generated from bulk RNA sequencing data to identify cell types from single-cell transcriptomes. An anchor graph is constructed to depict the relationship between reference samples and cells. By applying a connectivity constraint to the learned graph, LFSC enables the preservation of the underlying cluster structure. Moreover, the overall complexity of LFSC is linear to the size of the data, which greatly improves effectiveness and efficiency. By applying LFSC to real single-cell RNA sequencing datasets, we discovered that it has superior performance over existing baseline methods in clustering accuracy and robustness. An application using infiltrating T cells in liver cancer demonstrates that LFSC can successfully find new cell types, discover differently expressed genes, and explore new cancer-associated biomarkers.

## 1 Introduction

Bulk RNA sequencing (RNA-seq) technologies have been widely used to investigate gene expression patterns at the tissue level in recent decades (Conesa et al., 2016). However, they measure average global gene expression, which obscures true signals between heterogeneous cell types in the tissues. This technical limitation catalyzed the birth of single-cell RNA sequencing technology (scRNA-seq), which investigates RNA biology at the single-cell level. The transcriptome processes of humans and animals are highly heterogeneous; hence, it is more comprehensive and effective to study gene expression patterns using scRNA-seq (Li and Wang, 2021) in applications such as tumor heterogeneity (Bartoschek et al., 2018; Zhang et al.,. 2021a), disease diagnosis (Gate et al., 2020; Zakharov et al., 2020), and therapeutic treatment optimization (Zhang et al., 2020). The application of scRNA-seq data involves cell type identification (Aran et al., 2019; Wei and Zhang 2021), selection of differentially expressed genes (Sokolowski et al., 2021), cell-development trajectory construction (Liu et al., 2022), and cell–cell communication inferencing (Zhang et al., 2021b).

Among these applications, cell type identification is the most fundamental and essential. Traditional cell type identification methods consist of two steps: clustering cells using unsupervised learning algorithms and labeling cells based on specifically expressed marker genes in each cluster (Butler et al., 2018; Wang et al., 2018). While practical, these methods depend heavily on clustering performance and on prior knowledge of marker gene signatures, and they have high time complexity due to the calculated amounts of the cells’ similarity measurement stage. Based on labeled scRNA-seq data, researchers have proposed two types of cell type identification methods based on supervised learning. The first group of methods involves training a robust classifier on pre-labeled cells and then annotating other unlabeled cells with the trained classifier (Shao et al., 2021; Heydari et al., 2022). Another group of methods consists of two steps: embedding unlabeled cells into the subspace of labeled cells and then assigning the unlabeled cells according to the nearest neighbor-labeled cells (Pliner, Shendure, and Trapnell, 2019; Lotfollahi et al., 2021). Given the limitations to labeled scRNA-seq datasets, supervised methods cannot be widely used, especially for discovering rare cell populations (Qi et al., 2020).

Compared with scRNA-seq datasets, many bulk RNA-seq datasets have been archived in recent decades. Hence, some cell type identification methods integrating bulk RNA-seq datasets with known cell types have also been proposed recently. These methods attempt to use information from bulk RNA-seq data to annotate single-cell data. Specifically, they often identify cell types by correlating single-cell transcriptomes with reference datasets of pure cell types sequenced by RNA-seq, then iteratively improve the label inferences. SingleR (Aran et al., 2019) and RCA (Li et al., 2017) are the only two known methods of identifying cell types based on reference bulk RNA-seq data. However, it is difficult to detect subtle differences between cells using information from an external reference, since information from one sample in the bulk data comes from one tissue, while the information from the scRNA-seq data comes from one cell (Li and Wang, 2021).

**FIGURE 1:**
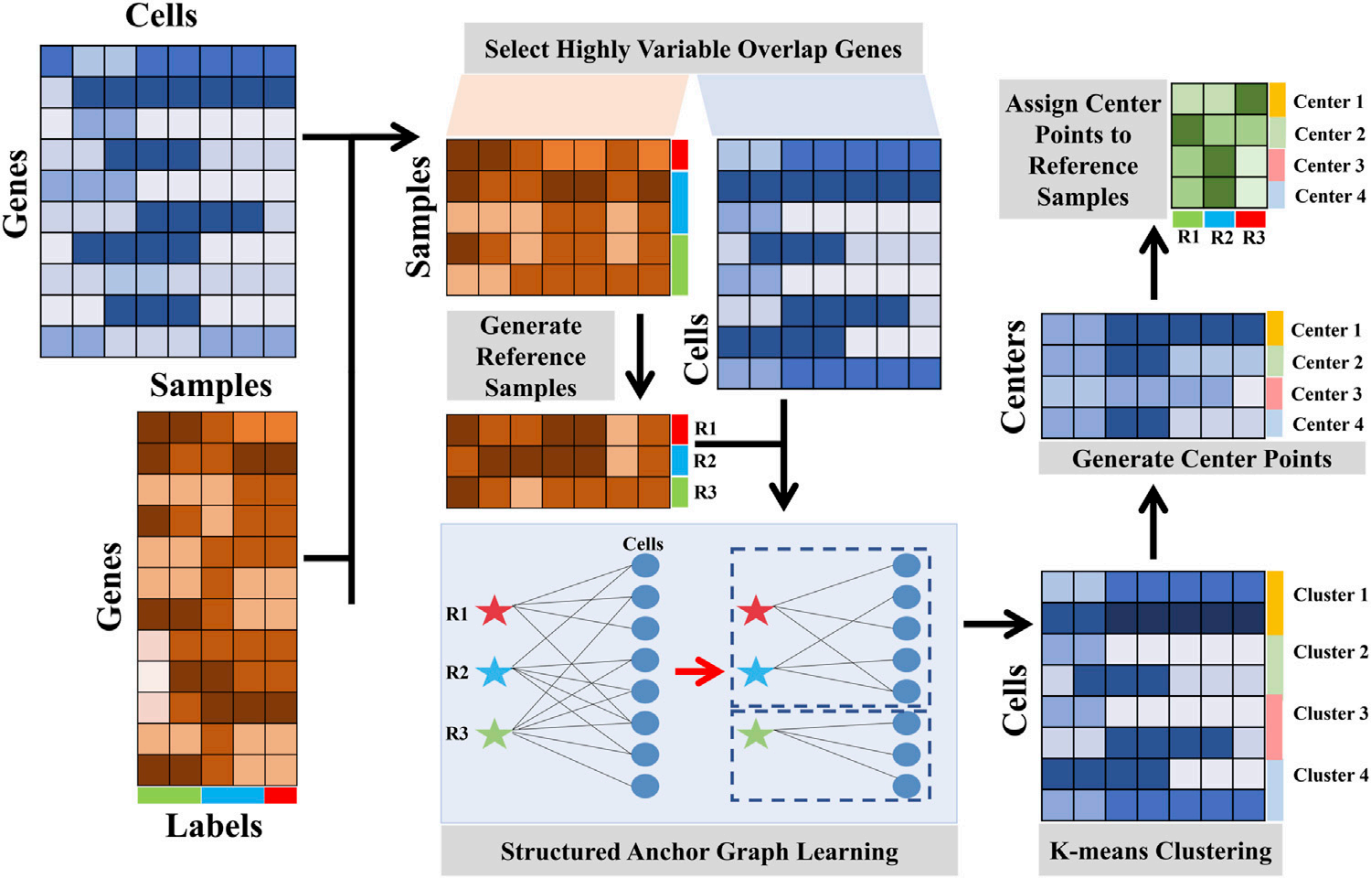
orkflow of the LFSC.

To address these issues, we present a linear fast semi-supervised clustering (LFSC) algorithm that integrates reference-bulk and single-cell transcriptome data using an anchor graph to improve the effectiveness and efficiency of clustering. The overview of LFSC is shown in Figure 1.

• Unlike SingleR and RCA, LFSC generates a dictionary matrix with *m* reference samples from bulk RNA-seq data or labeled scRNA-seq datasets by averaging gene expression profiles in the same cell type. Then, LFSC learns the relationship between cells in scRNA-seq data and the reference samples, generating an anchor graph with *k*-connected components, where *k* denotes the number of clusters.
• The advantages of LFSC are that 1) its affinity matrix, based on an anchor graph, preserves the underlying cluster structure of the data, which also reduces memory costs, and 2) its overall complexity is linear to the size of the data, which greatly improves effectiveness and efficiency.
• Through benchmark evaluations with 21 real scRNA-seq datasets and application to infiltrating T cells in liver cancer, we demonstrate that LFSC is superior to existing baseline methods.

## 2 Methods and materials

We consider LFSC to be different from supervised learning, in which the goal is to minimize one specific loss function, given the labels of samples. LFSC is also different from unsupervised learning because it is designed under weak supervision: a small set of bulk RNA-seq data, called reference samples, can represent the neighborhood structure of cells in scRNA-seq data. LFSC is regarded as a semi-supervised method since prior knowledge of referenced cell types, generated from bulk RNA-seq data, is combined in the unsupervised clustering process. The details of related studies and the LFSC method are provided in the following paragraphs.

### 2.1 Subspace clustering and anchor graph

Given a set of data $X\in R^{d\times n}$ , where *n* and *d* denote the number of samples and the number of features, respectively, subspace clustering assumes that data samples can be represented by a linear combination of samples underlying the same subspace. This means that X=XS , where the linear combination matrix $S\in R^{n\times n}$ can be modeled as the similarity graph among samples. To find the optimal solution of S , the estimating process is formulated as

$$\begin{array}{cc} \min_{S}{\left\|X-XS\right\|}^{2}+\delta f\left(S\right) & s.t.S\geq 0,S1=1, \end{array}$$ (1)

where δ>0 is a hyperparameter that balances the reconstruction error (first term) and the regularize function (second term f(∙) ). 1 denotes a column vector with all elements being one. The time complexity in solving Eq. 1 is 0 (*n* ^3^), which is costly in terms of running time and storage for large-scale data.

Anchor points, a small set of data samples, were selected to represent the landmarks of the data and preserve the underlying neighbor structure (see Supplementary Section S7). To reduce the computational sources, the anchor graph $A\in R^{n\times m}$ between anchor points and other data points was used in subspace clustering (Chen and Deng, 2011) as the anchor graph A is smaller than the similarity graph S . The estimating process is reformulated as

$$\begin{array}{cc} \min_{A}{\left\|X-ZA\right\|}^{2}+\delta f\left(A\right) & s.t.A\geq 0,A1=1 \end{array},$$ (2)

where Z denotes the dictionary matrix. Typically, the anchor points are selected by implementing the K-means algorithm on the dataset (Chen and Deng, 2011), and the centroid points in K-means are updated by calculating the average signals of samples in the same cluster. Hence, the average characteristic of the anchor points is naturally similar to that of the referenced bulk RNA-seq samples, which measure the average expression levels of specific genes in one tissue. We believe the anchor graph to be a potential tool for integrating reference bulk RNA-seq and scRNA-seq data.

### 2.2 Data preprocessing

In LFSC, the data preprocessing procedure includes two steps: quality control and normalization. First, data quality control is utilized to filter the low-expressed genes. If a gene has less than 5% or more than 95% of non-zero elements across all cells, it is filtered out. For data normalization, we utilize log-transform normalization, in which each element ( $m_{ij}$ ) of the expression profile *M* is transformed as follows:

$${\bar{m}}_{ij}=\log\left(\frac{m_{ij}\times 10000}{\sum_{i}m_{ij}}+1\right).$$ (3)

### 2.3 Selecting highly variable overlap genes

To reduce redundant features, we first identified the set of genes that were most variable in the expression profile, using the function *FindVariableFeatures* in the package *Seurat* (Butler et al., 2018). The details of selecting highly variable overlapping genes are provided in Supplementary Section S8. After selecting the highly variable genes, we then selected the genes that overlapped between the remaining genes in scRNA-seq data and the genes in bulk RNA-seq data.

### 2.4 Structured anchor graph learning

Constructing an affinity matrix among cells is the key step to identifying cell types in most computational approaches. In LFSC, we integrated reference bulk RNA-seq data and scRNA-seq data into the anchor graph with *k*-connected constraint, which not only improves the clustering performance but also reduces the computational sources. Structured anchor graph learning consists of two steps: generating reference samples with bulk RNA-seq data and constructing a structured anchor graph with reference samples. We used the scRNA-seq data matrix $X=\left(x_{1},\cdots ,x_{i},\cdots x_{n}\right)\in R^{m\times n}$ , with m highly variable genes and n cells, and the bulk RNA-seq data matrix $R=\left(r_{1},\cdots ,r_{j},\cdots r_{d}\right)\in R^{m\times d}$ , with m highly variable genes and d samples. Details of the structured anchor graph learning are provided in the following paragraphs.

#### 2.4.1 Generating reference samples from RNA-seq data

In LFSC, we must generate the reference samples which are regarded as anchor points in the anchor graph. Typically, the reference samples are generated from the bulk RNA-seq data. We calculated Pearson’s correlation coefficients $P\left(x_{i},r_{j}\right)$ between cell $x_{i}$ and bulk sample $r_{j}$ . We also generated the remaining sample set $R^{\prime }{\in R}^{m\times d^{\prime }}$ , the element having the greatest correlation, with at least one cell compared to other bulk samples:

$$R^{\prime }=\left\{r_{j}|\underset{r_{j}\in R}{argmax}P\left(x_{i},r_{j}\right)\;and\exists x_{i}\in X\right\}.$$ (4)

With the emergence of the labeled scRNA-seq data, LFSC also provides the option of generating reference samples from the scRNA-seq data. We generated one reference sample for each cell type by measuring the average expression profile for highly variable genes from cells in the same cell type.

#### 2.4.2 Constructing the structured anchor graph with reference samples

Given the scRNA-seq data $X=\left(x_{1},\cdots ,x_{i},\cdots x_{n}\right)\in R^{m\times n}$ and reference sample $R^{\prime }=\left(r_{1}^{\prime },\cdots ,r_{j}^{\prime },\cdots r_{d^{\prime }}^{\prime }\right)\in R^{m\times d^{\prime }}$ , we utilized the bipartite graph $B=\left[\begin{array}{cc} 0 & A\\ A^{T} & 0 \end{array}\right]\in R^{{(d}^{\prime }+n)\times {(d}^{\prime }+n)}$ to represent the anchor graph $A\in R^{d^{\prime }\times n}$ . The normalized Laplacian L is defined as

$$L=I-D^{-\left(\frac{1}{2}\right)}BD^{-\left(\frac{1}{2}\right)},$$ (5)

where $D\in R^{{(d}^{\prime }+n)\times {(d}^{\prime }+n)}$ is a diagonal matrix, the *i*th diagonal element of which is calculated as $d_{ii}=\overset{d^{\prime }+n}{\underset{j=0}{\sum }}b_{ij}$ . Chung and Graham (1997) have demonstrated that the normalized Laplacian L associated with non-negative matrix B has the following property.

Theorem 1 The number of connected components in the bipartite graph B is equal to the multiplicity *k* of the eigenvalue zero of the normalized Laplacian L.

Theorem 1 indicates that if $rank\left(L\right)=\left(d^{\prime }+n\right)-k$ , the bipartite graph B with $d^{\prime }$ reference samples and n cells can be clustered into *k* groups. Motivated by Theorem 1, we added a constraint to the clustering model, which is formulated as

$$\min_{A}{\left\|X-R^{\prime }A\right\|}^{2}+\delta {\left\|A\right\|}^{2},s.t.\;A\geq 0,A1=1,\;rank\left(L\right)=\left(d^{\prime }+n\right)-k.$$ (6)

As the rank constraint is hard to solve, we borrowed the idea from the related literature (Nie et al., 2019) to relax Eq. 6 as

$$\min_{A}{\left\|X-R^{\prime }A\right\|}^{2}+\delta {\left\|A\right\|}^{2}+\beta Tr\left(F^{T}LF\right),\;s.t.\;A\geq 0,A1=1,F^{T}F=I,$$ (7)

where $F\in R^{{(d}^{\prime }+n)\times k}.$ In LFSC, the problem (Eq. 7) can be solved by an alternating optimization method; more precisely, we solved A and F by fixing one solution and then updating the other one iteratively (see details in Supplementary Section S1).

#### 2.4.3 Estimating the cluster number *k*

Before the implementation of LFSC, we automatically estimated the cluster number *k* using the *R* package *clustree* (Zappia and Oshlack, 2018) with the default parameters. The details for estimating the number of clusters are provided in Supplementary Section S9. Finally, the clustering results were achieved by performing the K-means algorithm with the estimated cluster number *k*. The pseudo-code for LFSC is summarized in Algorithm 1.

#### 2.4.4 Identifying cell types *via* labeled transcriptomics data

In LFSC, we annotated cell types in scRNA-seq by calculating the Pearson coefficient between cell types from labeled transcriptomics data and cluster annotations of unknown scRNA-seq data. Based on the overlapping HVGs, the cell type of scRNA-seq data is annotated as

$${rc}_{i}=mean\left(x_{l}\right),\;s.t.\;x_{l}\in c_{i};$$

$$CT\left({rc}_{i}\right)=\left\{ \begin{array}{c} \begin{array}{cc} \left\{CT\left(r_{j}\right)|\underset{r_{j}\in R}{argmax}P\left({rc}_{i},r_{j}\right)\right\} & if\;P\left({rc}_{i},r_{j}\right)>0.6,\exists r_{j}\in R, \end{array}\\ \begin{array}{cc} Unknown\;type, & else\;P\left({rc}_{i},r_{j}\right)\leq 0.6,\forall r_{j}\in R; \end{array} \end{array}\right.$$ (8)

$$CT\left(x_{l}\right)=CT\left({rc}_{i}\right).$$

where $C=\left\{c_{1},{\cdots c}_{i},\cdots c_{K}\right\}$ is denoted as the K clustering results, and $c_{i}$ and ${rc}_{i}$ are the clustering index and the reference cell of cluster *i*, respectively. We defined CT(θ) as the cell type of the cluster or reference sample θ . The correlation analysis of overlapping variable genes between reference transcriptomics data and unlabeled scRNA-seq data was implemented to distinguish closely related cell types. The pseudo-code for LFSC is summarized in Algorithm 1.

### 2.5 Complexity analysis

In LFSC, we utilized an anchor graph to integrate the reference samples from bulk RNA-seq data and unlabeled cells from the scRNA-seq data, so that the complexity would reduce significantly. More precisely, we defined the number of iterations as t . In the alternating optimization method, we applied SVD on $A{\in R}^{d^{\prime }\times n}\left(d^{\prime }\ll n\right)$ to calculate the matrices U (taking $O({t(d^{\prime \prime }}^{3}+d^{\prime }n))$ ) and W (taking $O\left(td^{\prime }n\right)$ ). Using Supplementary Eq. 4, the problem can be efficiently solved in parallel using the MATLAB function *quadprog*, costing $O\left({td^{\prime }}^{3}n\right)$ . In addition, it costs $O\left(t^{\prime }{nk}^{2}\right)$ in applying K-means on U to obtain the clustering results, where $t^{\prime }$ denotes the number of iterations in K-means. Hence, the overall time complexity of the LFSC is linear to the number of cells n . For space complexity, in addition to commonly used sources like storing the scRNA-seq data O(mn) and bulk RNA-seq data $O\left(d^{\prime }n\right)$ , we need the storage sources $O\left(d^{\prime }n\right)$ for A , while that of the original graph A is $O\left(n^{2}\right)$ . In the alternating optimization method, the matrices B and D are stored as the sparse matrix, given their specific structures, while the space complexities of U, V and W are $O\left(nk\right),\;O\left(d^{\prime }k\right)$ and $O\left(d^{\prime }n\right)$ . Hence, the complete space complexity of LFSC also reduces significantly.

### 2.6 Evaluation metrics, test datasets, and baseline methods

We used the adjusted Rand index (ARI), accuracy (ACC), normalized mutual information (NMI), purity, and silhouette coefficient as our evaluation metrics (see details in Supplementary Section S2). We downloaded 21 public scRNA-seq datasets generated by four sequencing protocols (see details in Table 1) as the test datasets. We also selected six state-of-the-art methods (see details in Table 2 and Supplementary Section S3) as the compared baseline methods. In addition, we analyzed infiltrating T cells in liver cancer to examine LFSC’s application value in finding new cell types, discovering differently expressed genes, and exploring new cancer-associated biomarkers.

**TABLE 1:** Summary of the 21 real single-cell RNA-seq datasets.

Dataset	Cells	Genes	Types	Protocol	Resource	Usage	Confidence
Treutlin	80	23271	5	SMARTer	Human lung epithelium	Clustering	Silver standard
Yan	90	20214	7	Tang	Human preimplantation	Clustering and parameter analysis; visualization	Gold standard
Ting	114	14405	5	Drop-seq	Human circulating tumor	Clustering	Silver standard
mECS	182	8989	3	HiSeq	Mouse embryonic stem cells	Clustering	Silver standard
Buettner	189	8989	3	Drop-seq	Mouse T cells	Clustering	Silver standard
Goolam	214	41480	5	Smart-seq	Mouse embryonic cells	Clustering and parameter analysis; visualization	Gold standard
Ginhoux	251	11834	3	Smart-seq	Mouse conventional dendritic cells	Clustering	Silver standard
Deng	268	22431	7	Smart-seq	Mouse embryo cell	Clustering and parameter analysis; visualization	Gold standard
Pollen	301	23730	11	Smart-seq	Human cerebral cortex	Clustering and parameter analysis; visualization	Gold standard
Patel	430	5848	5	Smart-seq	Human glioblastomas	Clustering	Silver standard
Usoskin	622	17772	11	Drop-seq	Mouse lumbar cells	Clustering	Silver standard
Kolod	704	13473	3	SMARTer	Embryonic stem cells	Clustering	Silver standard
Seger	1099	25525	9	Smart-seq	Pancreatic islet	Clustering	Silver standard
Tasic	1679	24150	49	SMARTer	Mouse cortical cells	Clustering	Silver standard
Grun	1915	23536	3	CEL-seq	Hematopoietic stem cells	Clustering	Silver standard
Baron	1937	20125	14	InDrop	Pancreatic islet	Clustering	Silver standard
Zeisel	3005	19972	47	STRT-seq	Mouse cortex cells	Clustering	Silver standard
Marques	5053	23556	13	C1	Mouse neuronal cells	Clustering	Silver standard
Macosko	6418	23288	39	Drop-seq	Mouse retina cells	Clustering and running time analysis	Silver standard
Chen	14437	23284	45	Drop-seq	Hypothalamic cells	Clustering and running time analysis	Silver standard
Campbell	21086	26774	35	Drop-seq	Hypothalamic cells	Clustering and running time analysis	Silver standard

**TABLE 2:** Summary of the compared baseline methods.

Algorithm	Language	Theory	Link
SingleR	R	Semi-supervised	https://github.com/dviraran/SingleR
RCA	R	Semi-supervised	https://github.com/GIS-SP-Group/RCA
Garnett	R	Semi-supervised	https://github.com/cole-trapnell-lab/garnett
SC3	R	Unsupervised	https://github.com/hemberg-lab/SC3
Seurat	R	Unsupervised	https://github.com/satijalab/seurat
SIMLR	R	Unsupervised	https://github.com/BatzoglouLabSU/SIMLR

## 3 Results

### 3.1 LFSC outperforms six baseline methods for clustering single-cell transcriptomes

**FIGURE 2:**
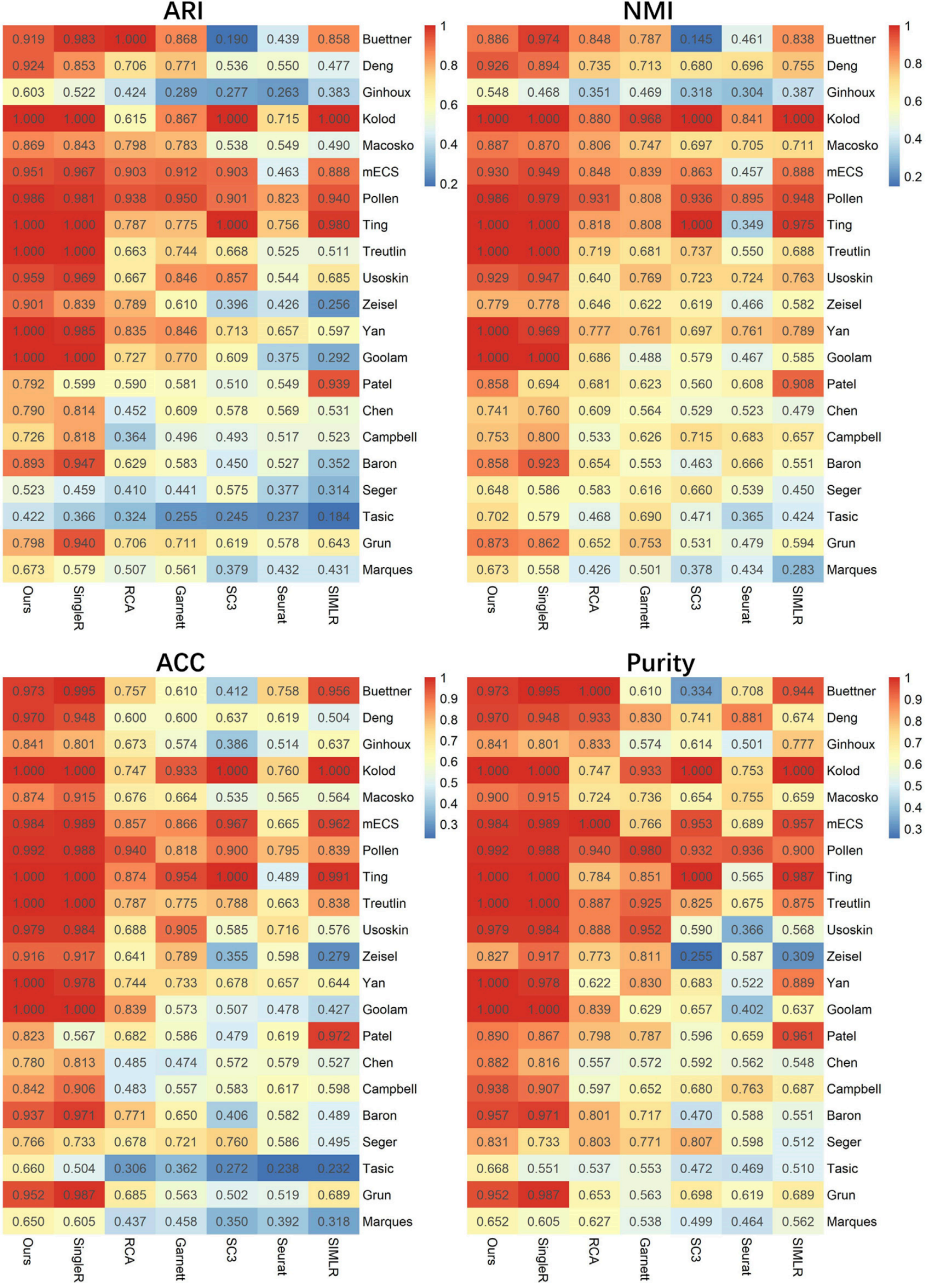
lustering evaluation (ARI, NMI, ACC, and purity) heatmap of LFSC and six baseline methods on 21 real scRNA-seq datasets.

To investigate the clustering performance of LFSC, we applied LFSC and six baseline methods on 21 real scRNA-seq datasets. The parameter settings of the six baseline methods and LFSC are provided in Supplementary Table S1. We also used the ARI, NMI, ACC, and purity metrics to evaluate the clustering results. For the scRNA-seq datasets, we generated reference samples by summing up the gene expression profiles in the same cell types, then averaging them with the number of cells (see details in Methods). The clustering results of LFSC and six baseline methods are presented in Figure 2 and Table 3. LFSC clearly improved clustering performance for 21 real scRNA-seq datasets. For example, LFSC obtained the optimal ARI solution for 13 out of 21 datasets, followed by SingleR (11 datasets) and SIMLR (one dataset). More precisely, LFSC obtained completely correct labels (ARI value equal to 1) on five datasets, followed by SingleR (four datasets). For NMI values, LFSC obtained the optimal solutions on 14 scRNA-seq datasets and the second-best solutions on seven datasets. For the other two clustering evaluation metrics, LFSC also had better clustering performance. In addition, LFSC statistically improved clustering performance on scRNA-seq datasets. As shown in Table 3, we applied statistically significant comparisons with the paired Wilcoxon signed-rank test. The symbol ≈ means that there was no significant difference between LFSC and the compared method; the symbol − means LFSC was worse than the compared method, and the symbol + denotes the opposite. The *p*-value was set as 0.05. The results demonstrate that LFSC is superior to the six baseline methods for four clustering evaluation metrics. The average ARI values in Table 3 show that LSFC (0.844) increased by about 1.5%, 21.9%, 24.3%, 29.8%, 38.6%, and 30.7% compared to SingleR (0.831), RCA (0.659), Garnett (0.679), SC3 (0.592), Seurat (0.518), and SIMLR (0.585). For NMI values, LFSC (0.856) was superior to SingleR (0.838), RCA (0.681), Garnett (0.685), SC3 (0.633), Seurat (0.570), and SIMLR (0.679). Similar conclusions can be drawn from the results of ACC and purity values. Furthermore, the semi-supervised clustering methods performed better than the unsupervised clustering methods. More precisely, the average ARI values of the semi-supervised clustering methods (0.753) were significantly better than those of the unsupervised clustering methods (0.565). To avoid the basis of comparing with average measurement, we also calculated the mean rank of four clustering evaluation metrics on the real scRNA-seq dataset (see Table 3). For ARI values, the mean rank of LFSC was 5.857, which was better than other baseline methods (SingleR: 5.524, RCA: 4.905, Garnett: 4.238, SC3: 3.381, Seurat: 1.667, and SIMLR: 2.429). For NMI values, LFSC produced the optimal mean rank value (5.476), followed by SingleR (5.333), RCA (4.476), and Garnett (4.524). For the other two clustering evaluation metrics, the mean rank values of LFSC were also better than those of the baseline methods. Based on the aforementioned discussion, we believe that LFSC significantly improves clustering.

**TABLE 3:** Clustering results of LFSC and baseline methods on real scRNA-seq datasets.

Metric		Ours	SingleR	RCA	Garnett	SC3	Seurat	SIMLR
ARI	Average	0.844	0.831	0.659	0.679	0.592	0.518	0.585
	Mean rank	5.857	5.524	4.905	4.238	3.381	1.667	2.429
	+/−/≈	N/A	11/4/7	21/0/1	22/0/0	19/2/1	22/0/0	20/1/1
NMI	Average	0.856	0.838	0.681	0.685	0.633	0.570	0.679
	Mean rank	5.476	5.333	4.476	4.524	4.000	1.857	2.333
	+/−/≈	N/A	12/4/6	22/0/0	22/0/0	20/2/0	22/0/0	20/1/1
ACC	Average	0.902	0.886	0.683	0.674	0.604	0.591	0.645
	Mean rank	5.714	4.810	4.667	4.762	4.048	1.714	2.286
	+/−/≈	N/A	10/4/8	22/0/0	22/0/0	20/2/0	22/0/0	20/1/1
Purity	Average	0.916	0.902	0.778	0.742	0.669	0.622	0.724
	Mean rank	5.571	5.048	4.952	4.714	3.333	2.095	2.286
	+/−/≈	N/A	11/4/7	20/0/2	22/0/0	20/2/0	22/0/0	20/1/1

### 3.2 Robustness analysis of highly confident datasets

**FIGURE 3:**
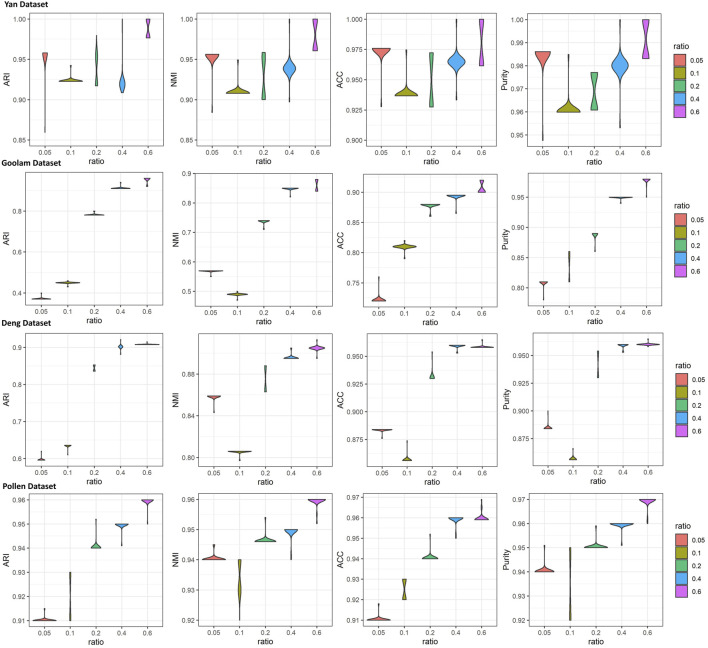
iolin plots of clustering evaluations (ARI, NMI, ACC, and purity) of LFSC with different downsampling ratios for four highly confident scRNA-seq datasets.

**FIGURE 4:**
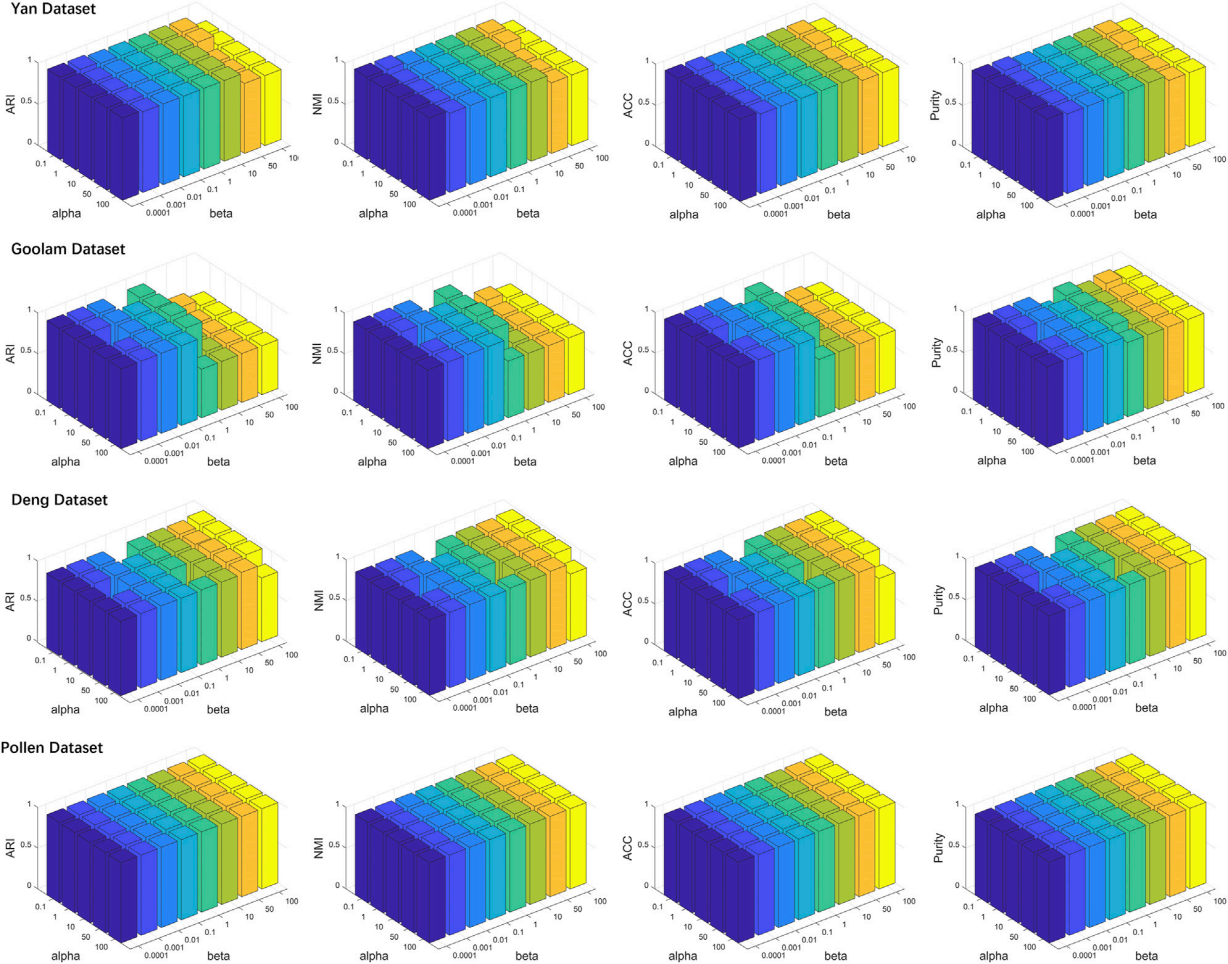
lustering evaluation (ARI, NMI, ACC, and purity) results of LFSC with different hyperparameters on four highly confident scRNA-seq datasets.

To investigate the effects of generated reference samples on clustering performance, we introduced the downsampling strategy to generate reference samples with different sampling ratios (see details in Supplementary Section S4). Since the cell types of some real scRNA-seq datasets were generated by computational approaches (Kiselev et al., 2017), the highly confident scRNA-seq datasets, called gold standard datasets, were selected as the test datasets, including Yan, Goolam, Deng, and Pollen (see details in Table 1). The downsampling ratios were set as 0.05, 0.1, 0.2, 0.4, and 0.6. For different ratios, we implemented LFSC with randomly generated reference samples 30 times and calculated four clustering evaluation metrics. Figure 3 shows how the clustering performance of LFSC varied with different downsampling ratios. We found that the clustering performance of LFSC gradually improved by increasing the ratio from 0.05 to 0.6. This demonstrates that more selected samples may generate more representative reference samples. In addition, to investigate the effects of hyperparameter (alpha: *α* and beta: *β*) settings on clustering performance, we implemented LFSC with different combinations of hyperparameters on highly confident scRNA-seq datasets. Alpha (*α*) was selected as 0.1, 1, 10, 50, and 100. Beta (*β*) was selected as 0.0001, 0.001, 0.01, 0.1, 1, 10, 50, and 100. Figure 4 shows how the clustering performance of LFSC varied with different combinations of *α* and *β*. We found that the performance of LFSC is stable for a large range of *α* and *β* values. In practice, we recommend optimizing *α* and *β* by fixing *α* and tuning *β*.

### 3.3 LFSC speed improves significantly for large scRNA-seq datasets

**FIGURE 5:**
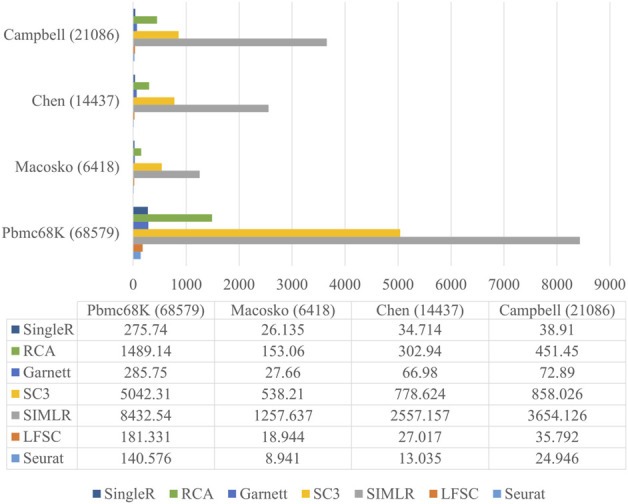
Running time of LFSC and six baseline methods on datasets Macosko, Chen, Campbell, and Pbmc68K.

Since the number of cells sequenced with advanced sequencing protocol is growing, it is important to have a satisfactory running time when analyzing scRNA-seq data. To demonstrate that LFSC is efficiently implemented in practice, we compared the running time between LFSC and six baseline methods on four relatively large real scRNA-seq datasets using a six-core and 32 GB memory computer. The Macosko, Chen, Campbell, and Pbmc68K datasets contain 6418, 14437, 21086, and 68579 cells, respectively. Figure 5 shows that Seurat (Macosko: 8.491s, Chen: 13.035s, Campbell: 24.946s, Pbmc68K: 140.576s) was the fastest of the seven methods, followed by LFSC (Macosko: 18.944s, Chen: 27.017s, Campbell: 35.792s, Pbmc68K: 181.331s) and SingleR (Macosko: 26.135s, Chen: 34.714s, Campbell: 38.91s, Pbmc68K: 275.74s). Furthermore, LFSC was comparable with RCA (Macosko:153.06s, Chen: 302.94s, Campbell: 451.45s, Pbmc68K: 1489.14s) and Garnett (Macosko: 27.66s, Chen: 66.98s, Campbell: 72.89s, Pbmc68K: 285.75s). SC3 and SIMLR cost significantly more time than the other five methods. Although Seurat is superior in running time to LFSC, SingleR, RCA, and Garnett, it is the closest method, especially when Seurat has applied a parallelization operator that is lacking in LFSC.

### 3.4 The visualization ability of LFSC

**FIGURE 6:**
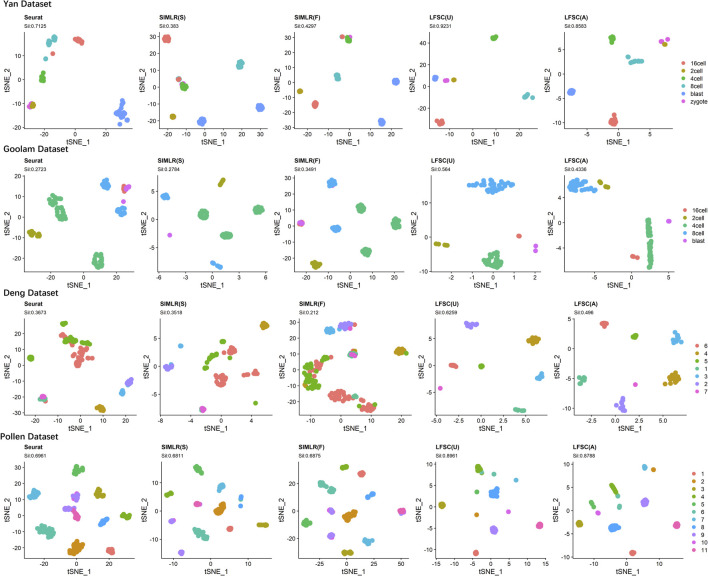
t-SNE plots of LFSC and baseline methods with corresponding silhouette coefficients and clustering results on four highly confident scRNA-seq datasets.

We investigated LFSC’s ability to embed cells into the two-dimensional space using *t*-distributed stochastic neighbor embedding (t-SNE). In the experiment, we first chose four real gold standard scRNA-seq datasets (shown in Table 2). Then, we selected Seurat and SIMLR as compared baseline methods for LFSC since they are graph-based models. In particular, SIMLR and LFSC are variants of spectral clustering, which generates the affinity and decomposition matrixes in the clustering process. Thus, we implemented t-SNE on the generated matrixes for SIMLR and LFSC (see Figure 6). The silhouette coefficient (Sil, see Supplementary Section S2) is a widely used cluster metric that compares inter- and intra-distances among data points based on the clustering partition. To measure the quality of the visualization, we used the silhouette coefficient to analyze whether cells of the same types were closer, while those from different cell types were more separated in the t-SNE space. Figure 6 shows the visualization plots of LFSC, Seurat, and SIMLR for the four highly confident scRNA-seq datasets. For the dataset Yan, we found that LFSC(U) and LFSC(A) produced the best (Sil: 0.9231) and the second-best (Sil: 0.86) performances, respectively, which were significantly better than Seurat, SIMLR(S), and SIMLR(F). Similar conclusions were obtained for the dataset Goolam, where the Sil values of LFSC(U), LFSC(A), Seurat, SIMLR(S), and SIMLR(F) were 0.564, 0.4236, 0.2723, 0.2784, and 0.3491. It is noted that only LFSC identified the correct number of cell types (five clusters, see Figure 6), while Seurat and SIMLR clustered cells into six and seven clusters, respectively. In the dataset Deng, LFSC was not only superior to compared methods in Sil values and clustering but also had the best performance in visualization since LFSC separated cells from different cell types and combined cells from the same cell types. In the dataset Pollen, only LFSC detected the correct number of clusters (see Figure 6), and the learned embedding space of LFSC was well separated, while cells in the same cell type were more compact. In conclusion, the aforementioned analysis indicates that LFSC has better visualization ability than do the compared baseline methods.

### 3.5 Ablation study of LFSC

We completed the ablation study to investigate the importance of each component in LFSC. In particular, the ablation experiment was designed as follows. 1) Without HVG selection, all overlap genes between the scRNA-seq data and the reference transcriptomics data were selected. 2) Without reference transcriptomics data, we generated the reference sample by directly implementing the K-mean algorithm on the scRNA-seq data. Supplementary Table S2 summarizes the NMI, ARI, ACC, and purity values for the 21 scRNA-seq datasets. In the first step, the HVG selection had a positive impact on clustering performance, which not only reduced redundant features but also sped up the convergence process. Second, without the reference transcriptomics data, the clustering performance significantly deteriorated for the 21 scRNA-seq datasets, which demonstrates that the reference transcriptomics data are important for LFSC to improve robustness and efficiency. In conclusion, the aforementioned analysis indicates that all components in LFSC were designed effectively and reasonably.

### 3.6 LFSC detected two new function-specific subtypes of tumor-infiltrating lymphocytes

**FIGURE 7:**
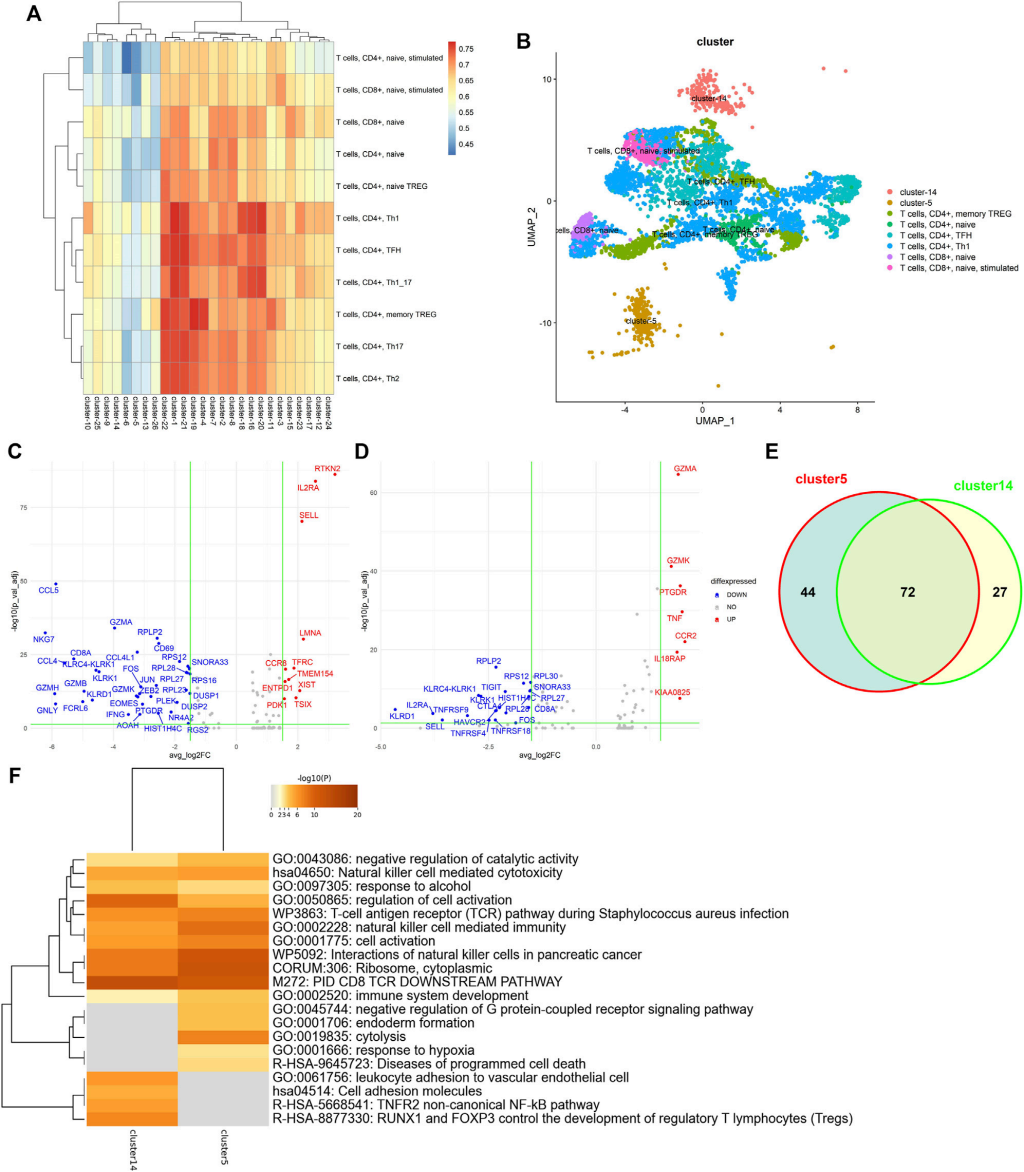
Single-cell transcriptional profiling of the tumor-infiltrating lymphocytes. **(A)** Heatmap of Pearson coefficient values between 26 clusters and 11 T-cell subtypes; **(B)** t-SNE plots of tumor-infiltrating lymphocytes annotated by LFSC using the ImmGen database as the reference; **(C)** volcano plot showing differentially expressed genes in cluster 5; **(D)** volcano plot showing differentially expressed genes in cluster 14; **(E)** Venn diagram showing the overlap of DEGs between cluster 5 and cluster 14; **(F)** heatmap of the enriched term across DEGs on cluster 5 and cluster 14.

Exploring subtypes of the tumor-infiltrating lymphocytes is a benefit for the investigation of immunotherapies and associated clinical responses in cancers. To investigate the exploring ability in biological analysis, we downloaded a GEO dataset (GSE98638) containing 5,063 single T cells isolated from peripheral blood, tumor, and adjacent normal tissues from six hepatocellular carcinoma patients (Zheng et al., 2017). We first implemented *clustree* (Zappia and Oshlack 2018) to explore the correct number of clusters. In a cluster tree plot, one node represents a cluster, and a larger node means the cluster has more data points. Since the tree has no branches and the leaf nodes have similar sizes (see Supplementary Figure S1), when the number of clusters equaled 26, the stability and robustness of clustering were best. Then, we used the Immunological Genome Project (ImmGen) database (Aran et al., 2019) as the reference bulk RNA-seq data and applied LFSC with the number of clusters equal to 26. The heatmap of the Pearson coefficient between the 26 clusters and 11 T-cell subtypes is shown in Figure 7A. Cluster 5 and cluster 14 were significantly unassociated with the 11 T-cell subtypes. We annotated the other 24 clusters with the 11 T-cell subtypes by maximizing the associated coefficient values (see Figure 7B). Cluster 5 and cluster 14 were well separated from other clusters, which indicates that they are different from the six annotated T-cell subtypes in function or biological process.

To determine the biological differences between cluster 5 and cluster 14, we applied Seurat to identify the differentially expressed genes (DEGs) of cluster 5 and cluster 14. The DEG genes were selected under the criteria 1) absolute log2-fold change larger than 1.5, and 2) adjusted *p*-value of F test <0.05. There were 48 DEGs (11 upregulated genes and 37 downregulated genes for cluster 5 (see Figure 7C) and 28 DEGs (7 upregulated genes and 21 downregulated genes; see Figure 7D) for cluster 14. Supplementary Figure S2 shows the t-SNE projection of the tumor-infiltrating lymphocytes to be colored by DEGs of cluster 5 (*RTKN2*, *IL2RA*, *SELL*, *LMNA*, *TFRC*, and *CCR8*) and cluster 14 (*GZMA*, *GZMK*, *PTGDR*, *TNF*, *CCR2*, and *IL18RAP*). Some genes are associated with immunological diseases. For example, *CCR2* and *CCR8* are protein-coding genes associated with diseases including human immunodeficiency virus type 1 and molluscum contagiosum. *GZMA* and *GZMK* are well-known marker genes regarded as T-cell- and natural killer cell-specific serine proteases. Some studies have demonstrated *IL2RA* and *IL18RAP* to be associated with the same cytokine signaling pathway in the immune system.

To further demonstrate that cluster 5 and cluster 14 have specific functions, we completed functional enrichment analysis on the DEGs with the analysis tool *Metascape* (Zhou et al., 2019). As seen in Figure 7F, we found that the most enriched functions for cluster 5 and cluster 14 were enriched for different biological terms. For example, cluster 5 enriched for the term GO: 0019835, which is related to the rupture of cell membranes and the loss of cytoplasm, and GO: 0002520, which is associated with immune system development. Although cluster 14 is also enriched in the same terms, like GO: 0002520, GO:0050865 (regulation of cell activation), and GO: 0001775 (cell activation), DEGs of cluster 14 were significantly enriched in the biological process of leukocyte adhesion to vascular endothelial cells (GO:0061756) and the development of regulatory T lymphocytes (R-HSA-8877330). This proves that the newly detected cluster 5 and cluster 14 have different functions in biological processes.

### 3.7 LFSC detects DEGs associated with biomarkers of liver cancer

**FIGURE 8:**
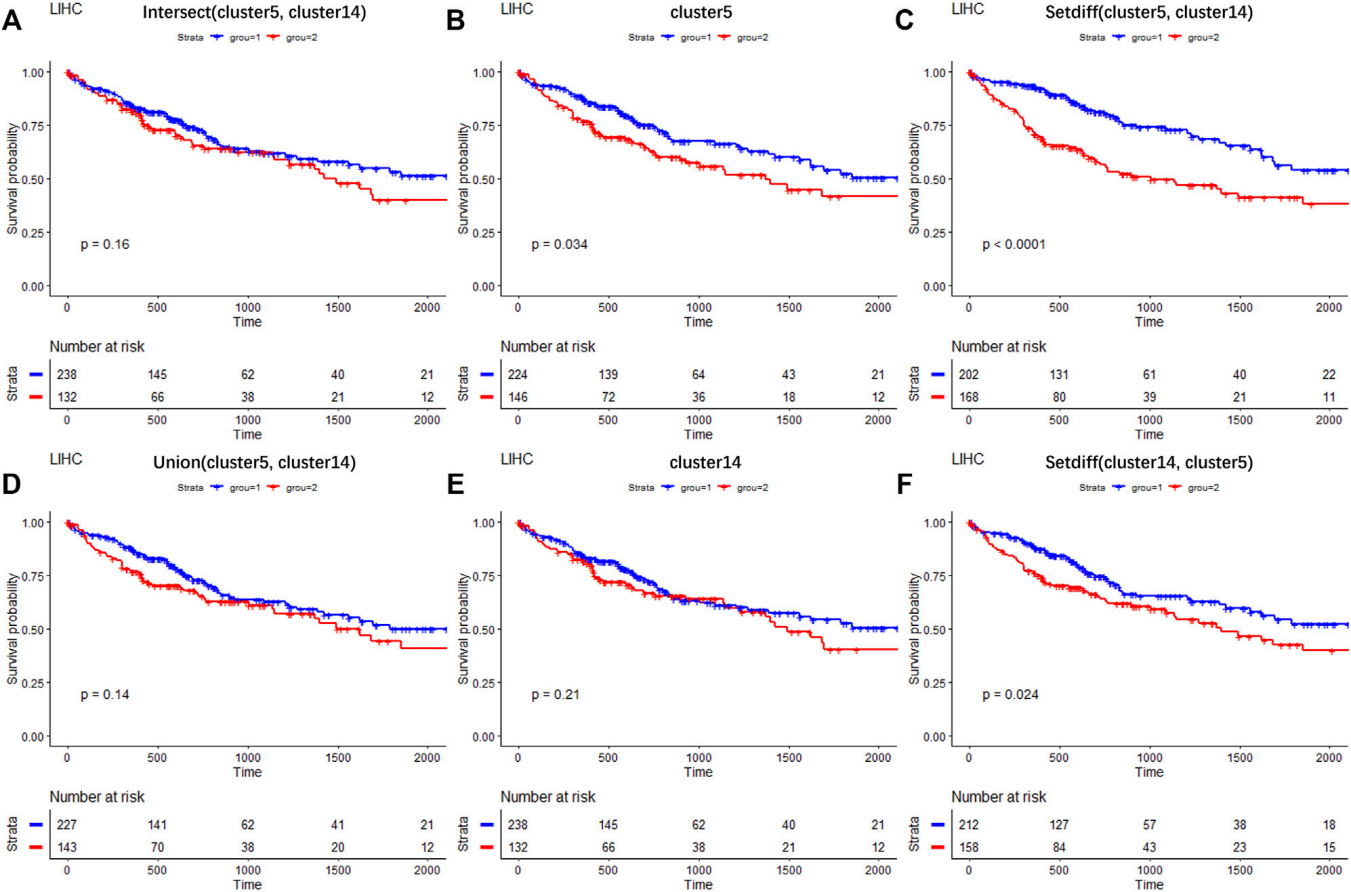
Results of survival analysis on selected DEGs across TCGA LIHC clinical data. Survival curves on the intersection of DEGs between cluster 5 and cluster 14 **(A)**; on DEGs of cluster 5 **(B)**; on DEGs belonging to cluster 5 but not to cluster 14 **(C)**; on the combination of DEGs between cluster 5 and cluster 14 **(D)**; on DEGs of cluster 14 **(E)**; on DEGs belonging to cluster 14 but not to cluster 5 **(F)**.

To investigate the clinical research values of selected DEGs of newly found subtypes (cluster 5 and cluster 9), liver hepatocellular carcinoma (LIHC) samples from The Cancer Genome Atlas Program (TCGA) dataset (Tomczak et al., 2015) were used to test the correlations between selected genes and patient survival. The analysis details are provided in Supplementary Section S5. Figure 8 shows the survival curves for the DEGs of cluster 5 and cluster 14. Three hundred and seventy LIHC tumor samples were divided into two groups based on the expression profiles of six gene sets composed of different combinations of DEGs. Significantly, the intersection set of the DEGs on cluster 5 and cluster 14 (*p* = 0.16, paired Wilcoxon test, Figure 8A), the combined set of these (*p* = 0.14, paired Wilcoxon test, Figure 8D), and the DEGs of cluster 14 (*p* = 0.21, paired Wilcoxon test, Figure 8E) were statistically unassociated with poor prognosis. Meanwhile, the DEGs of cluster 5 (*p* = 0.034, paired Wilcoxon test, Figure 8B) and cluster-specific DEGs (paired Wilcoxon test, *p* < 0.0001, Figure 8C and *p* = 0.024, Figure 8D) correlated with good prognosis in TCGA cohort. Thus, our results provide evidence that the DEGs of newly found clusters are biomarkers in the tumor microenvironment of LIHC.

Algorithm 1 Framework of LFSC

*Input*:
 scRNA-seq dataset: X ;
 Reference dataset: R

 Hyperparameters: δ and β

*Begin*
1. ***Step 1*:** Implement quality control and Normalize data X using (3);
2. ***Step 2*:** Identify highly variable overlap genes with the package *Seurat* on X and R ;
3. ***Step 3*:** Estimate the cluster number, *k*, using the package *clustree* on X ;
4. ***Step 4*:** Generate the reference samples $R^{\prime }$ with reference dataset R using (4);
5. ***Step 5*:** Construct the structured anchor graph A with reference samples $R^{\prime }$ ;
6.  ***Step 5.1*:** Initialize the matrix F ;
7.  **While** convergence condition does not meet **do**
8.  ***Step 5.2*:** Update A in Supplementary Eq. S4 using convex quadratic programming;
9.  ***Step 5.3*:** Update U in Supplementary Eq. S7 by Supplementary Lemma S1;
10.  *end while*
11. ***Step 6*:** Run K-means on with the cluster number *k*;
*End*
*Output:*
 Clustering result Y .

## 4 Conclusion

We presented a linear fast semi-supervised clustering method, based on bulk and single-cell transcriptomes, that has the following characteristics: 1) LFSC generates reference samples with bulk-RNA-seq or labeled single-cell RNA-seq data, which implicitly provides the label information to the graph construction process; 2) LFSC introduces anchor graph theory to measure the similarities between unlabeled cells and a small number of reference samples, which significantly reduces the size of the graph; and 3) the *K*-connectivity constraint is added to the cell-reference anchor graph to preserve the underlying clustering structure of the data. In general, the proposed mechanisms not only improve the clustering accuracy of the model but also make its overall complexity linearly related to data size, and they reduce the memory overhead of the model.

The experiments on several scRNA-seq datasets demonstrate the following conclusions: 1) LFSC is superior to state-of-the-art methods in clustering accuracy and robustness; 2) the visualization analysis proves that the anchor graph in LFSC can retain the correct clustering structure of the data, and the learning embedding space has good separation, which has a better visualization effect compared with the benchmark methods; and 3) the results of ablation analysis show that all components of LFSC are effective and reasonable. In addition, the case study of infiltrating T cells in liver cancer demonstrated that LFSC shows promising application potential in discovering new cell types, identifying differentially expressed genes, and exploring new cancer-related biomarkers.

## Data Availability

The original contributions presented in the study are included in the article/Supplementary Material, and further inquiries can be directed to the corresponding author.
