# Supplementary material for: LFSC: A linear fast semi-supervised clustering algorithm that integrates reference-bulk and single-cell transcriptomes
Source: Front Genet. 2022 Dec 1;13:1068075. doi: 10.3389/fgene.2022.1068075 (PMC9754124; doi:10.3389/fgene.2022.1068075)
Supplement: Supplementary file 1 [file Table1.DOCX]

# Supplementary Materials

**1 Alternating optimization method in the LFSC**

In LFSC, problem (7) in the main manuscript can be solved by an alternating optimization method, more precisely, we solve $\mathbb{A}$ and $\mathbb{F}$ by fixing one solution and then updating the other one iteratively.

1. Fix$\mathbb{F}$and Update$\mathbb{A}$.

As$B$and$\mathbb{D}$are associated with$\mathbb{A}$, the problem (7) in the main manuscript is reformulated as

$\min_{\mathbb{A}} \sum_{i}^{n} \sum_{j}^{d^{'}} \mathbb{a}_{ij}\left\| x_{i}-{r^{'}}_{j} \right\|^{2}+\delta\left\| \mathbb{A} \right\|^{2}+\beta Tr\left( \mathbb{F}^{T}L\mathbb{F} \right), s.t. \mathbb{A}\geq0,\mathbb{A}\boldsymbol{1}=\boldsymbol{1.}$ (1)

In addition, we have the following equation:

$Tr\left( \mathbb{F}^{T}L\mathbb{F} \right)=\sum_{i}^{n+d^{'}} \sum_{j}^{{n+d}^{'}} \frac{1}{2}b_{ij}\left\| \frac{\mathbb{f}_{i}}{\sqrt{\mathbb{d}_{i}}}-\frac{\mathbb{f}_{j}}{\sqrt{\mathbb{d}_{j}}} \right\|^{2}.$ (2)

According to the structure of$B$, equation (2) can be rewritten as

$Tr\left( \mathbb{F}^{T}L\mathbb{F} \right)=\sum_{i}^{n} \sum_{j}^{d^{'}} \frac{1}{2}\mathbb{a}_{ij}\left\| \frac{\mathbb{f}_{i}}{\sqrt{\mathbb{d}_{i}}}-\frac{\mathbb{f}_{n+j}}{\sqrt{\mathbb{d}_{n+j}}} \right\|^{2}.$ (3)

We define${w_{ij}=\left\| \frac{\mathbb{f}_{i}}{\sqrt{\mathbb{d}_{i}}}-\frac{\mathbb{f}_{n+j}}{\sqrt{\mathbb{d}_{n+j}}} \right\|}^{2}$and problem (1) can be rewritten as

$\min_{\mathbb{A}} \sum_{i}^{n} \sum_{j}^{d^{'}} (\mathbb{a}_{ij}\left\| x_{i}-{r^{'}}_{j} \right\|^{2}+\delta{\mathbb{a}_{ij}}^{2}+\beta\mathbb{a}_{ij}w_{ij}), s.t. \mathbb{A}\geq0,\mathbb{A}\boldsymbol{1}=\boldsymbol{1.}$ (4)

Problem (4) can be solved using convex quadratic programming.

1. Fix$\mathbb{A}$and Update$\mathbb{F}$.

When$\mathbb{A}$is fixed, the first two terms of the problem (7) in the main manuscript are constant. Problem (7) in the main manuscript is reformulated as

$\max_{\mathbb{F}} Tr\left( \mathbb{F}^{T}\mathbb{D}^{-(1/2)}B\mathbb{D}^{-(1/2)}\mathbb{F} \right), s.t. \mathbb{F\in}\mathcal{R}^{{(d}^{'}+n)\times k},\mathbb{F}^{T}\mathbb{F=}I\boldsymbol{.}$ (5)

Considering the specific structure of$B$, we rewrite$\mathbb{F}$and$\mathbb{D}$as,

$\mathbb{F=}\left[ \begin{matrix} U \\ V \end{matrix} \right]\mathbb{, D=}\left[ \begin{matrix} \mathbb{D}_{U} & \\ & \mathbb{D}_{V} \end{matrix} \right]$, (6)

where$U\in\mathcal{R}^{n\times k}, V\in\mathcal{R}^{d^{'}\times k}, \mathbb{D}_{U}\in\mathcal{R}^{n\times n}$, and $\mathbb{D}_{V}\in\mathcal{R}^{d^{'}\times d^{'}}$. Problem (5) is reformulated as

$\max_{U^{T}U+V^{T}V=I} Tr\left( U^{T}{\mathbb{D}_{U}}^{-(1/2)}B{\mathbb{D}_{V}}^{-(1/2)}V \right)$, (7)

Problem (7) can be solved according to the following lemma [1].

*Lemma* 1: *Suppose*$Z\in\mathcal{R}^{n\times m}, X\in\mathcal{R}^{n\times k}$, and$Y\in\mathcal{R}^{m\times k}$. *The optimal solutions to the problem*

$$\max_{X^{T}X+Y^{T}Y=I} Tr\left( X^{T}ZY \right),$$

*are*$X=(\frac{\sqrt{2}}{2})U_{1}$*and*$Y=(\frac{\sqrt{2}}{2})V_{1}$, *where*$U_{1}$*and*$V_{1}$ *are the top k left and right singular vectors of*$Z$, *respectively*.

**2 Definitions of the used evaluation metrics**

In this section, we provide the definitions of the Adjust Rand Index (ARI), Accuracy (ACC), Normalized Mutual Information (NMI), Purity, and Silhouette Coefficient.

**2.1 Adjusted Rand Index (ARI)**

Adjust Rand Index (ARI) is another important external metric, which evaluates on a pairwise-basis about whether data points are incorrectly grouped. ARI can be described as follows,

$$ARI(X,Y)=\frac{\sum_{p=1}^{|Y|} \sum_{q=1}^{|X|} C_{N_{pq}}^{2}-a_{3}}{(a_{1}+a_{2})/2-a_{3}}\text{，}$$

$$a_{1}\text{=}\sum_{i=1}^{|Y|} C_{N_{i}}^{2}\text{，} a_{2}\text{=}\sum_{i=1}^{|X|} C_{N_{j}}^{2}, a_{3}\text{=}\frac{2\times a_{1}a_{2}}{N(N-1)}.$$

where *N_i_* denotes the number of data points in cluster *C_i_*, *N_j_* denotes the number of data points belonging to *C_j_* and *N_ij_* denotes the number of points in the consistency between *C_i_* and *C_j_*.

**2.2 Accuracy (ACC)**

Accuracy is a widely used external metric, which is defined as,

$$ACC(X,Y)=\frac{\sum_{i} \alpha\left(map(l_{i})=y_{i}\right)}{N}.$$

where *y*_i_ and *x*_i_ denote the true label and predicted label of sample *i*. *α*(•) denotes the indicator function. *x*_i_ is mapped to the best cluster center with the Kuhn-Munkres algorithm.

**2.3 Normalized Mutual Information**

Normalized Mutual Information (NMI) is a widely used external metric in machine learning, which uses information-theoretical to measure the correlation between candidate results and true labels. It can be expressed as follows,

$$\text{NMI}(X,C)=\frac{\sum_{p=1}^{|C|} \sum_{q=1}^{|X|} |C_{p}\cap X_{q}|\log\frac{n|C_{p}\cap X_{q}|}{|C_{p}\text{|} \times\text{|} X_{q}|}}{max(-\sum_{p=1}^{|C|} |C_{p}\text{|} \log\frac{|C_{p}|}{n},-\sum_{q=1}^{|X|} |X_{q}|\log\frac{|X_{q}|}{n})}\text{，}$$

where *X* and *C* represent the clustering candidate and the ground truth on the set of *n* data points, respectively, while |*X*| is the number of clusters and |*C*| is the number of classes.

**2.4 Purity**

The Purity Index is another important metric in clustering analysis, which is defined as,

$$Purity(X,Y)=\frac{\sum_{i} max_{j}\left|X_{i}\cap Y_{j}\right|}{N}.$$

where *X* and *Y* denote the true label and predicted label, respectively.

**2.5 Silhouette Coefficient**

The silhouette Coefficient is a useful metric for evaluating clustering performance. We assume a data set with *N* samples could be divided into *k* clusters *C*_i_ (*i* = 1, 2, … , *k*). For each cluster, the silhouette indicator is defined as follows,

$Sil\left( i \right)=\frac{1}{n_{i}}\sum_{m=1}^{n_{i}} \frac{[b\left( m \right)-a(m)]}{max[b\left( m \right),a\left( m \right)]}$,

where$n_{i}$is the number of samples in cluster *C*_i_. *a*(*m*) is the average distance between sample *m* in cluster *C*_i_ and other samples in cluster *C*_j_. *b*(*m*) denotes the minimum average distance between sample point *m* in cluster *C*_j_ to all samples in another cluster *C*_i_. The average silhouette indicator of all samples is defined as follows,

$Sil=\frac{1}{k}\sum_{i=1}^{k} Sil\left( i \right)$.

**3 Baseline methods**

We selected 6 representative cell type identification methods as the compared baseline methods, including 3 semi-supervised learning-based methods (SingleR [2], RCA [3] and Garnett [4]) and 3 unsupervised learning-based methods (SC3 [5], Seurat [6] and SIMLR [7]).

For the semi-supervised learning-based methods, SingleR and RCA are annotating the scRNA-seq data by reference to bulk transcriptomes. More precisely, they assign cell types for single-cell transcriptomes by comparing reference bulk tissue RNA-seq data sets with pure cell types. In Garnett, authors utilized the marker information to select cells and then used them to train the regression-based classifier for annotating cell types.

For the unsupervised learning-based methods, SC3 is a clustering ensemble framework that can integrate different clustering results based on Euclidean similarity, Pearson correlation as well as Spearman correlation. Seurat is the most widely used scRNA-seq data analysis tool, which performs dimension reduction, clustering, and visualization in turn by learning a similarity measure using graph theory. In SIMLR, authors proposed a novel framework to learn a cell-to-cell similarity measure from expression data observed for heterogeneous samples.

**4 The downsampling strategy**

Since LFSC generates the reference samples by measuring the average expression profile on highly variable genes of cells in the same cell type, we introduce the downsampling strategy to generate different reference samples and investigate the effects on clustering performance. We set the sampling ratio as 0.05, 0.1, 0.2, 0.4, and 0.6, and then randomly selected cells in each cell type according to the sampling ratio. Finally, we generated the reference sample for each cell type by the average expression profile of highly variable genes of selected cells in the same cell type.

**5** **TCGA data analysis**

The TCGA data of Liver Hepatocellular Carcinoma (LIHC) samples were used to test the correlation between selected genes and patient survival. According to the DEGs from cluster 5 and cluster 14, we generated six gene subsets, including the union set of DEGs between two clusters, DEGs that belong to cluster 5 not belong to cluster 14, DEGs that belong to cluster 14 not belong to cluster 5, the intersected DEGs between two clusters, DEGs of cluster 5 and DEGs of cluster 14. The LIHC samples were clustered into two groups based on the expression of six gene subsets, respectively. With the clinical survival data of LIHC samples, the survival analysis was performed by the *R* package *survival*, while survival curves were fitted by the *survfit* function and the difference between the two groups was tested by *survdiff* function.

**6 t-distributed Stochastic Neighbor Embedding (t-SNE) and Uniform Manifold Approximation and Projection (UMAP)**

The t-distributed Stochastic Neighbor Embedding (t-SNE) is a nonlinear statistical method for embedding high-dimensional data for visualization in a low-dimensional space [8]. In t-SNE, it first constructs a probability distribution that similar objects are assigned a higher probability while dissimilar ones are assigned a lower probability. Second, t-SNE defines a similar probability distribution over the points in the subspace, and it minimizes the KL divergence between the two distributions with respect to the locations of the points in the subspace.

The Uniform Manifold Approximation and Projection (UMAP) is also a nonlinear dimensionality reduction technique, which is designed based on the assumption that the data is uniformly distributed on a locally connected Riemannian manifold and that the Riemannian metric is locally constant or approximately locally constant [9].

**7 The anchor graph clustering**

Graphs are structures consisting of a number of data nodes and a set of edges connecting different nodes. Graph Clustering (GC) is the method that groups the data nodes of the graph into clusters where edges are relatively closed. In the past few years, many researchers have made efforts to construct an effective graph to improve the clustering performance [10-13]. In scRNA-seq data clustering, there are also some impressive works in identifying cell subtypes, such as Seurat [6], SIMLR [7], SinNLRR [14], SMSC [15], MPSSC [16], and so on. To well integrate effective information and deal with the scalability issue in clustering, anchor graph clustering has been introduced, where selects a small number of anchor points to construct a small and tractable graph for reducing the computational burden and improving the clustering performance[17]. For example, Qiang proposed a fast clustering algorithm based on representative anchors to solve the spectral clustering problem directly with a small time cost [18]. Lin et al. proposed a graph clustering algorithm with a smooth representation of selected anchor points. They designed a new regularization term to explore the high-order neighborhood information [19]. To address the separation problem between anchors sampling and clustering, Sun et al.propoesd a unified framework combining the achor learning process and graph construction process [20]. These works have demonstrated anchor graph-based clustering algorithms are superior to the original graph clustering algorithms in computational complexity, especially in handling the large-size datasets [21].

**8 The process of selecting highly variable genes**

In LFSC, the process of selecting highly variable genes consists of three steps:

- Firstly, the mean and a dispersion measure (variance/mean) for each gene across all cells are calculated;
- Next, each gene is placed into 20 bins according to the average expression across all cells. The function then applies z-score normalization on the dispersion measure of all genes within the bin;
- Finally, according to the parameter z-score cutoff, significantly variable genes are selected in descending order.

**9 The process of estimating** **the number of clusters *K***

In LFSC, the process of estimating the number of cluster *K* also consists of three steps:

- Firstly, LFSC clusters dataset with any clustering algorithm at different resolutions which can generate a set of nodes representing clusters;
- Next, the overlap between clusters at adjacent resolutions is used to build edges and the resulting graph is presented as a clustering tree.
- As the clustering trees show relationships between clusters and the edges in a clustering tree show how samples move between clusters as the resolution changes, the fewer edges and the larger size of clusters mean the clustering results are more stable and robust.

In general, the number of clusters of the most stable clustering results in the clustering trees is set as the number of clusters *K* in LFSC.

**Reference**

1. Nie, F., C.-L. Wang, and X. Li, *K-Multiple-Means*, in *Proceedings of the 25th ACM SIGKDD International Conference on Knowledge Discovery & Data Mining*. 2019. p. 959-967.

2. Aran, D., et al., *Reference-based analysis of lung single-cell sequencing reveals a transitional profibrotic macrophage.* Nature Immunology, 2019. **20**(2): p. 163-172.

3. Li, H., et al., *Reference component analysis of single-cell transcriptomes elucidates cellular heterogeneity in human colorectal tumors.* Nat Genet, 2017. **49**(5): p. 708-718.

4. Pliner, H.A., J. Shendure, and C. Trapnell, *Supervised classification enables rapid annotation of cell atlases.* Nat Methods, 2019. **16**(10): p. 983-986.

5. Kiselev, V.Y., et al., *SC3: consensus clustering of single-cell RNA-seq data.* Nat Methods, 2017. **14**(5): p. 483-486.

6. Butler, A., et al., *Integrating single-cell transcriptomic data across different conditions, technologies, and species.* Nat Biotechnol, 2018. **36**(5): p. 411-420.

7. Wang, B., et al., *SIMLR: A Tool for Large-Scale Genomic Analyses by Multi-Kernel Learning.* Proteomics, 2018. **18**(2): p. 1700232.

8. Maaten, L.v.d. and G. Hinton, *Visualizing data using t-SNE.* Journal of machine learning research, 2008. **9**(Nov): p. 2579-2605.

9. Becht, E., et al., *Dimensionality reduction for visualizing single-cell data using UMAP.* Nature biotechnology, 2019. **37**(1): p. 38-44.

10. Liu, X., et al., *Efficient and Effective Regularized Incomplete Multi-view Clustering.* IEEE Trans Pattern Anal Mach Intell, 2020.

11. Hu, Z., et al., *Multi-view spectral clustering via integrating nonnegative embedding and spectral embedding.* Information Fusion, 2020. **55**: p. 251-259.

12. Zhou, H., et al., *Identify ncRNA subcellular localization via graph regularized k-local hyperplane distance nearest neighbor model on multi-kernel learning.* IEEE/ACM Trans Comput Biol Bioinform, 2021. **PP**.

13. Wang, Z., et al., *Local structured feature learning with dynamic maximum entropy graph.* Pattern Recognition, 2021. **111**.

14. Zheng, R., et al., *SinNLRR: a robust subspace clustering method for cell type detection by nonnegative and low rank representation.* Bioinformatics, 2019. **35**(19): p. 3642--3650.

15. Qi, R., et al., *A spectral clustering with self-weighted multiple kernel learning method for single-cell RNA-seq data.* Briefings in Bioinformatics, 2020: p. 1-11.

16. Park, S. and H. Zhao, *Spectral clustering based on learning similarity matrix.* Bioinformatics, 2018. **34**(12): p. 2069-2076.

17. Chen, M.-S., et al., *Representation Learning in Multi-view Clustering: A Literature Review.* Data Science and Engineering, 2022. **7**(3): p. 225-241.

18. Qiang, Q., et al. *Fast multi-view discrete clustering with anchor graphs*. in *Proceedings of the AAAI Conference on Artificial Intelligence*. 2021.

19. Lin, Z. and Z. Kang. *Graph Filter-based Multi-view Attributed Graph Clustering*. in *IJCAI*. 2021.

20. Sun, M., et al., *Scalable Multi-view Subspace Clustering with Unified Anchors*, in *Proceedings of the 29th ACM International Conference on Multimedia*. 2021. p. 3528-3536.

21. Chen, X. and D. Cai. *Large scale spectral clustering with landmark-based representation*. in *Twenty-fifth AAAI conference on artificial intelligence*. 2011.

Figures


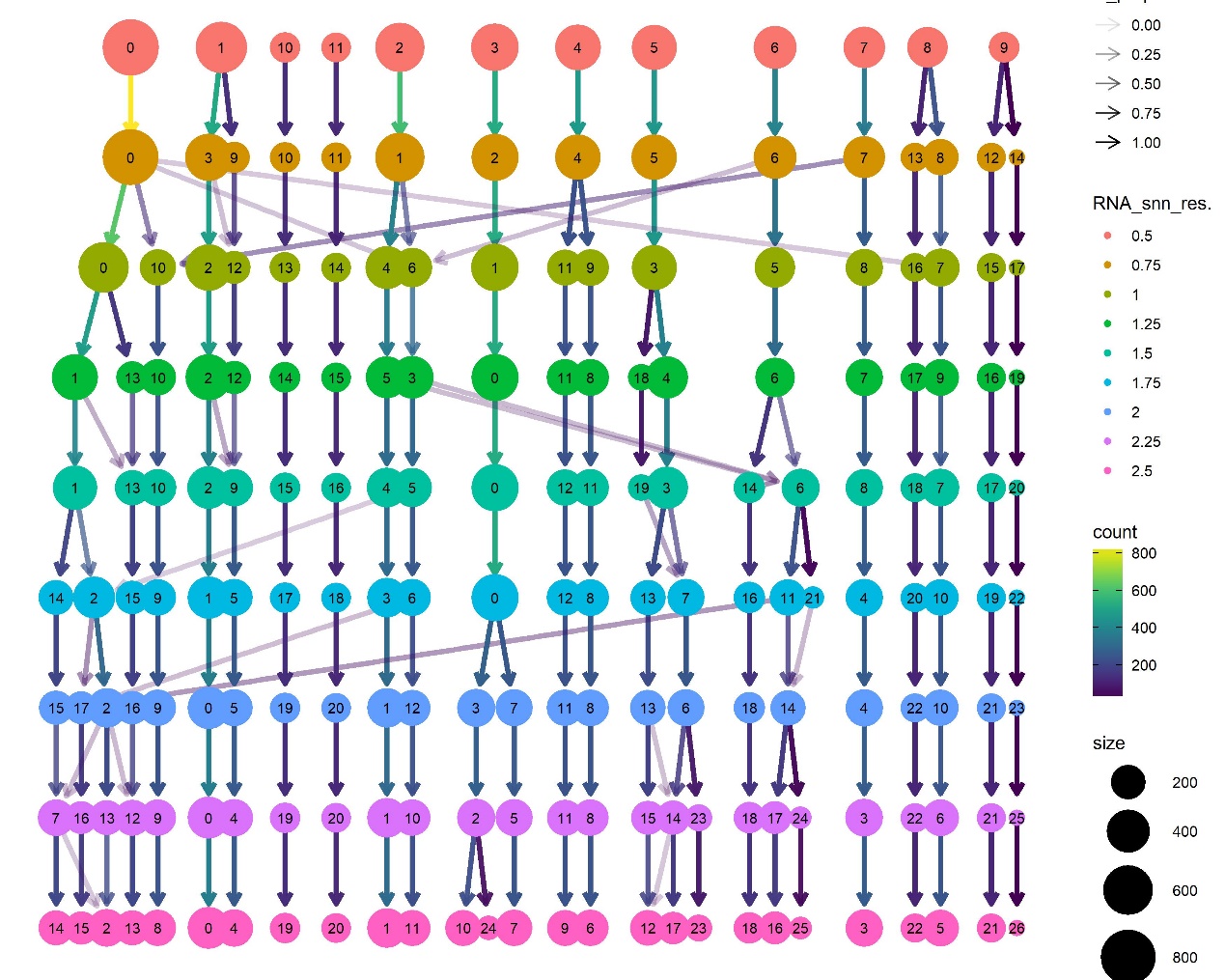


**Figure S1**. The cluster tree plot produced by *clustree* on the tumor-infiltrating lymphocytes.


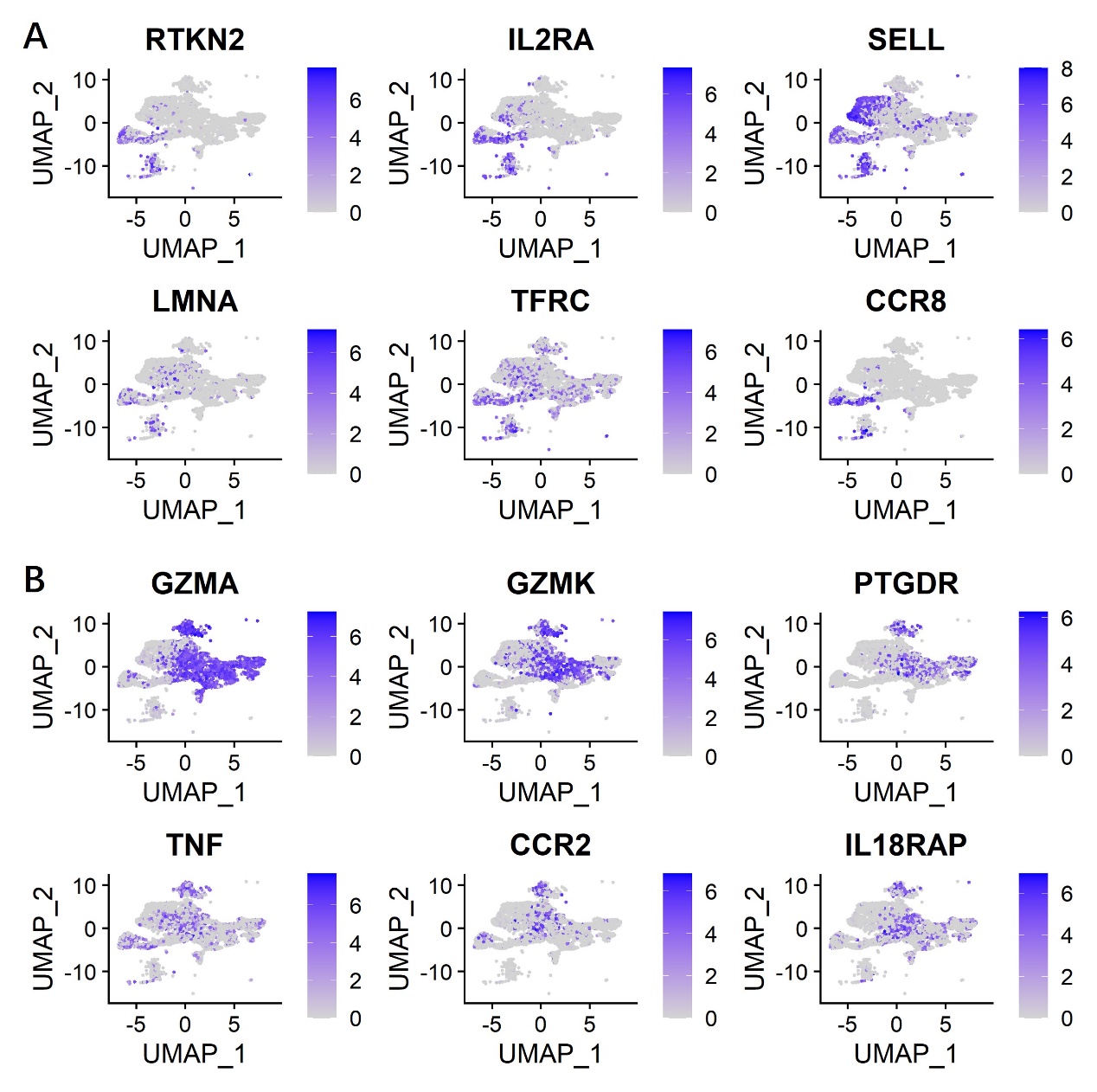


**Figure S2**. The t-SNE projection of the tumor-infiltrating lymphocytes colored by selected DEGs of cluster 5 (A) and cluster 14 (B).

Tables

**Table S1**. The parameter of LFSC and compared baseline methods.

| Method | Default parameters |
| --- | --- |
| SingleR | *quantile =* 0.8, *# The quantile of the correlation distribution.*  *tune.thresh =* 0.05, *# Threshold for maximum correlation to use in fine-tuning* |
| RCA | *k* = 10, *# Number of nearest neighbors.*  *threshold =* 0.1, *# The FDR threshold.* |
| Garnett | *num_unknown =* 500, # *Number of unknown cells compared against.* |
| SC3 | *svm_max =* 5000, # *The maximum number of cells below which SVM is not run*  *kmeans_iter_max =* 1e+09, # *The maximum number of iterations* |
| Seurat | *resolution =* 0.8, *# The resolution parameter. n.iter =* 10, *# Maximal number of iterations per random start*  *n.start* = 10, *# Number of random starts.*  *scale.factor* = 10000, *# The scale factor for cell-level normalization.* |
| SIMLR | *k =* 10*, # The tuning parameter.*  *kk =* 100, *# The number of principal components to be assessed in the PCA alpha* |
| LFSC | *δ=* 0.1, *# Hyperparameter Alpha.*  *β=* 1e-04, # *Hyperparameter Beta.* |

**Table S2**. The clustering results of ablation study on real scRNA-seq datasets.

| Dataset | Method | ACC | NMI | Purity | ARI |
| --- | --- | --- | --- | --- | --- |
| Treutlin | Without HVGs selection | 0.875 | 0.809 | 0.854 | 0.524 |
|  | Without reference transcriptomics data | 0.638 | 0.651 | 0.800 | 0.462 |
|  | LFSC | **1.000** | **1.000** | **1.000** | **1.000** |
| Yan | Without HVGs selection | 0.877 | 0.865 | 0.917 | 0.812 |
|  | Without reference transcriptomics data | 0.767 | 0.831 | 0.867 | 0.721 |
|  | LFSC | **1.000** | **1.000** | **1.000** | **1.000** |
| Ting | Without HVGs selection | 0.984 | 0.923 | 0.984 | 0.951 |
|  | Without reference transcriptomics data | 0.935 | 0.902 | 0.935 | 0.906 |
|  | LFSC | **1.000** | **1.000** | **1.000** | **1.000** |
| mECS | Without HVGs selection | 0.978 | 0.904 | 0.978 | 0.934 |
|  | Without reference transcriptomics data | 0.604 | 0.351 | 0.610 | 0.332 |
|  | LFSC | **0.984** | **0.930** | **0.984** | **0.951** |
| Buettner | Without HVGs selection | 0.935 | 0.830 | 0.932 | 0.901 |
|  | Without reference transcriptomics data | 0.632 | 0.417 | 0.643 | 0.409 |
|  | LFSC | **0.973** | **0.886** | **0.973** | **0.919** |
| Goolam | Without HVGs selection | 0.858 | 0.884 | 0.953 | 0.811 |
|  | Without reference transcriptomics data | 0.726 | 0.698 | 0.911 | 0.606 |
|  | LFSC | **1.000** | **1.000** | **1.000** | **1.000** |
| Ginhoux | Without HVGs selection | 0.832 | 0.448 | 0.732 | 0.513 |
|  | Without reference transcriptomics data | 0.729 | 0.368 | 0.729 | 0.421 |
|  | LFSC | **0.841** | **0.548** | **0.841** | **0.603** |
| Deng | Without HVGs selection | 0.826 | 0.878 | 0.893 | 0.824 |
|  | Without reference transcriptomics data | 0.681 | 0.729 | 0.748 | 0.461 |
|  | LFSC | **0.970** | **0.926** | **0.970** | **0.924** |
| Pollen | Without HVGs selection | 0.940 | 0.968 | 0.968 | 0.960 |
|  | Without reference transcriptomics data | 0.912 | 0.929 | 0.924 | 0.906 |
|  | LFSC | **0.992** | **0.986** | **0.992** | **0.986** |
| Patel | Without HVGs selection | 0.753 | 0.720 | 0.747 | 0.634 |
|  | Without reference transcriptomics data | 0.661 | 0.665 | 0.663 | 0.546 |
|  | LFSC | **0.823** | **0.858** | **0.890** | **0.792** |
| Usoskin | Without HVGs selection | 0.924 | 0.801 | 0.924 | 0.857 |
|  | Without reference transcriptomics data | 0.958 | 0.885 | 0.958 | 0.927 |
|  | LFSC | **0.979** | **0.929** | **0.979** | **0.959** |
| Kolod | Without HVGs selection | 0.954 | 0.977 | 0.958 | 0.961 |
|  | Without reference transcriptomics data | 0.938 | 0.908 | 0.916 | 0.906 |
|  | LFSC | **1.000** | **1.000** | **1.000** | **1.000** |
| Seger | Without HVGs selection | 0.675 | 0.636 | 0.783 | 0.481 |
|  | Without reference transcriptomics data | 0.530 | 0.524 | 0.829 | 0.347 |
|  | LFSC | **0.766** | **0.648** | **0.831** | **0.523** |

**Table S2**. The clustering results of ablation study on real scRNA-seq datasets (continued).

| Dataset | Method | ACC | NMI | Purity | ARI |
| --- | --- | --- | --- | --- | --- |
| Tasic | Without HVGs selection | 0.657 | 0.643 | 0.587 | 0.416 |
|  | Without reference transcriptomics data | 0.232 | 0.410 | 0.261 | 0.131 |
|  | LFSC | **0.660** | **0.702** | **0.668** | **0.422** |
| Grun | Without HVGs selection | 0.891 | 0.462 | 0.891 | 0.586 |
|  | Without reference transcriptomics data | 0.579 | 0.764 | 0.804 | 0.540 |
|  | LFSC | **0.952** | **0.873** | **0.952** | **0.798** |
| Baron | Without HVGs selection | 0.812 | 0.787 | 0.960 | 0.763 |
|  | Without reference transcriptomics data | 0.649 | 0.717 | 0.958 | 0.625 |
|  | LFSC | **0.937** | **0.858** | **0.957** | **0.893** |
| Zeisel | Without HVGs selection | 0.847 | 0.752 | 0.847 | 0.788 |
|  | Without reference transcriptomics data | 0.824 | 0.699 | 0.788 | 0.734 |
|  | LFSC | **0.916** | **0.779** | **0.827** | **0.901** |
| Marques | Without HVGs selection | 0.561 | 0.576 | 0.606 | 0.414 |
|  | Without reference transcriptomics data | 0.548 | 0.526 | 0.579 | 0.376 |
|  | LFSC | **0.650** | **0.673** | **0.652** | **0.673** |
| Macosko | Without HVGs selection | 0.831 | 0.841 | 0.825 | 0.777 |
|  | Without reference transcriptomics data | 0.550 | 0.723 | 0.771 | 0.469 |
|  | LFSC | **0.874** | **0.887** | **0.900** | **0.869** |
| Chen | Without HVGs selection | 0.723 | 0.739 | 0.870 | 0.663 |
|  | Without reference transcriptomics data | 0.514 | 0.616 | 0.841 | 0.500 |
|  | LFSC | **0.780** | **0.741** | **0.882** | **0.790** |
| Campbell | Without HVGs selection | 0.684 | 0.665 | 0.917 | 0.457 |
|  | Without reference transcriptomics data | 0.504 | 0.577 | 0.904 | 0.559 |
|  | LFSC | **0.842** | **0.753** | **0.938** | **0.726** |
